# Costs of testing sick children in primary care with pulse oximetry: Evidence from four countries, both with and without electronic clinical decision support

**DOI:** 10.1371/journal.pgph.0004644

**Published:** 2025-07-01

**Authors:** Susan Horton, Ulrich Adombi, Fenella Beynon, Mira Emmanuel-Fabula, Tara Herrick, Sandeep Kumar, Suzan Makawia, Mercy Mugo, Michael Onah, Michael Ruffo, Shally Awasthi, Maymouna Ba, Leah F. Bohle, Silvia Cicconi, Hélène Langet, Papa Moctar Faye, Honorati Masanja, Andolo Miheso, Deusdedit Mjungu, James Machoki M’Imunya, Ousmane Ndiaye, Kovid Sharma, Valérie D’Acremont, Kaspar Wyss, TIMCI Collaborator Group

**Affiliations:** 1 School of Public Health Sciences, University of Waterloo, Waterloo, Canada; 2 Faculté de médecine, Université Cheikh Anta Diop, Dakar, Sénégal; 3 Swiss Centre for International Health, Swiss Tropical and Public Health Institute, Basel, Switzerland; 4 University of Basel, Basel, Switzerland; 5 PATH, Geneva, Switzerland; 6 PATH, Seattle, Washington, United States of America; 7 PATH, New Delhi, India; 8 Ifakara Health Institute, Dar-es-Salaam, Tanzania; 9 Department of Economics and Development Studies, University of Nairobi, Nairobi, Kenya; 10 RTI, Research Triangle Park, North Carolina, United States of America; 11 Department of Pediatrics, King George’s Medical University, Lucknow, Uttar Pradesh, India; 12 PATH, Dakar-Fann, Senegal; 13 PATH, Dar-es-Salaam, Tanzania; 14 PATH, Nairobi, Kenya; 15 Faculty of Medicine, University of Nairobi, Nairobi, Kenya; 16 Centre for primary care and public health (Unisanté), Université de Lausanne, Lausanne, Switzerland; University College London, UNITED KINGDOM OF GREAT BRITAIN AND NORTHERN IRELAND

## Abstract

Introducing pulse oximeters (PO) at primary care facilities can help health workers identify severely ill children who need referral to hospital thereby allowing for improved child clinical outcomes. Adding clinical decision support algorithms (CDSA) can improve adherence to Integrated Management of Childhood Illness guidelines. The current study analyses the costs of introducing PO either with or without an electronic CDSA using an RCT in India and Tanzania and in a pre-post design with an electronic CDSA in Kenya and Senegal. The impact of the intervention is discussed for the RCT (trial registration NCT04910750) and for the pre-post study (trial registration NCT05065320), following SPIRIT guidelines. Economic data were collected in all four countries using questionnaires administered at primary health facilities and referral hospitals and supplemented by information from administrative sources, following CHEERS guidelines. Trained research assistants at the facilities collected data on children enrolled and health outcomes. Net costs per 100 children managed using PO ranged from $16.62 (Kenya, health center) to $70.51 (Tanzania, dispensary), in both cases using CDSA. Senegal was an outlier at $385.45, using PO and CDSA in the smaller postes de santé. Major causes explaining variation included training modality, numbers of sick children attending the facility, and the effect of PO and CDSA on use of antibiotics, diagnostics, and hospitalizations. Standard care (without PO) was associated with fewer severe complications (primarily untimely hospitalizations), at lower cost, in the two countries where effectiveness data are available, India and Tanzania. Scaling up PO use at primary care level nationally could have an important budgetary impact. Findings suggest ways that costs could potentially be reduced. However, hospitalization costs borne by households may affect both household and provider behavior and limit the potential clinical benefits of pulse oximetry.

## Introduction

Five million children under five died in 2021 across the globe [UNICEF, 2023] [1]. For children over the age of one month, infections are the main direct cause of mortality, with pneumonia being the largest single cause, accounting for almost three-quarters of a million under-five deaths globally in 2019 [2]. 80% of global under-five child deaths occur in sub-Saharan Africa and the Southern Asia subregion. According to WHO [2], there are low-cost, low-tech interventions for diagnosis and treatment of pneumonia. Diagnosing children such that they receive appropriate care for severe disease including pneumonia is a priority for helping to achieve the Sustainable Development Goals for reducing child mortality.

Measurement of oxygen saturation of blood can help identify children with severe disease who are missed when relying only on clinical signs [3–5]. Pulse oximeters (PO) are non-invasive devices which allow the identification of hypoxaemia. Clinical decision support algorithms (CDSAs) have been recommended by WHO to assist healthcare providers in following guidelines such as Integrated Management of Childhood Illness (IMCI) [6]. These technologies are not, as yet, widely available or used at primary care levels in sub-Saharan Africa and South Asia.

The TIMCI project undertook a multi-method impact evaluation through a pragmatic cluster randomised controlled trial (RCT) in India and Tanzania, and quasi-experimental pre-post studies in Kenya and Senegal, described in more detail in the study protocol [7], which also includes a summary health economics analysis plan. The project studied the introduction of PO with and without CDSA in four countries, with the aim of examining feasibility, acceptability, clinical impact and cost. The India RCT had two arms (PO/control), while the Tanzania RCT had three (PO with CDSA/PO without CDSA/control), while the pre-post studies both introduced PO with CDSA. The electronic CDSA was customized for each country to support international and national guidelines for care of sick children. A rich dataset using both qualitative and quantitative data was collected, and the cost analysis was one of several complementary analyses ongoing and completed. The impacts in the RCT countries (India and Tanzania) are described in [8], and the impacts in the pre-post countries (Kenya and Senegal) are described in [9]. The present study discusses the cost results for all four countries.

A search of PubMed (see S1 File) on cost and/or cost-effectiveness of introducing PO at primary care level in low- and middle-income countries (LMICs) identified three previous studies. A study of Thailand models the cost per DALY (disability-adjusted life-year) averted by introducing PO in primary care and concludes that PO both saves money and improves health outcomes [10]. The authors make the strong assumption that all of children identified with severe pneumonia adhere to the recommendation for referral to hospital. A study of 15 low- and middle-income countries models the introduction of PO and concludes that the cost per DALY averted ranges from $2.97 to $52.92 [11]. A study using data from an RCT from Ethiopia models the additional cost per case of severe pneumonia identified in the PO arm compared to standard care as $29 [5]. The study does not attempt to estimate cost per DALY averted, because the sample size (1804) is too small.

The cost-effectiveness component of the TIMCI project originally aimed to estimate the cost of using PO, with or without CDSA, as well as the cost per severe outcome averted, from the perspective of the health service provider. Severe complications included death and hospitalization without referral, or delayed hospitalization (occurring neither on the day of the consultation nor the following day). The comparator was usual care, namely consultation using neither PO nor CDSA. Since severe complications did not decrease with the intervention [8,9], cost-effectiveness was not calculated.

The four countries included in the TIMCI project represent a variety of contexts. There are differences across countries in care offered at facilities at different levels. More basic care generally includes outpatient care, preventive and pharmacy services and sometimes laboratory services. More advanced care can include limited inpatient beds for deliveries and clinics for TB, HIV, diabetes and hypertension. In one of the four countries (Senegal) only the more basic primary facilities (“postes de santé” or PS) were included, as PO were already in use at most of health centres (“centres de santé”) which host a broader primary care service package. In India, only rural primary care facilities were included, namely Primary Health Centres (PHC) and Community Health Centres (CHC). In Kenya and Tanzania both more basic and more advanced primary care facilities (dispensaries and health centres – HC – respectively) were included, in both rural and urban areas. In Kenya dispensaries are also referred to as level 2 facilities, and health centres as level 3 facilities.

There were also differences as to how and which healthcare providers received IMCI refresher training and were trained to use the PO, and the associated workflow implications. In all countries except India the training was on-site, while in India the training was organised on-line. In India, only medical doctors trained in allopathic (western) medicine are permitted to practice western medicine, while AYUSH practitioners (ayurvedic, yoga, unani, siddha and homeopaths) are not. While AYUSH practitioners may have participated in the training, they are not currently permitted to practice using PO. In the three sub-Saharan African countries nurses, nurse-midwives, clinical officers and medical officers undertake consultations; Kenya also utilizes a triage system in some facilities operated by nurses who can use PO, while other countries primarily use PO during the clinical consultation itself.

In the framework of the present study household costs incurred were considered only at hospital level, since these directly affect household adherence to recommendations for referral, and hence clinical outcomes. Costs to patients affect behavior. In all four countries treatment costs at primary level for children under five for key infectious diseases and conditions such as malnutrition are free or very nominal, as long as the necessary medicines and essential diagnostic tests are available at the public facilities. The same is true for hospitalization costs, although in the three sub-Saharan African countries appropriate referral procedures must be followed [12–14]. In India no referral from primary care is required to receive care at public hospitals [15].

In India perceptions of quality lead those who can afford private care to exercise that option: 62% of hospital beds are private [16]. In Kenya, around 30–40% of hospital beds in the country are private, some for-profit, others faith-based [13]. In Senegal, children under five receive free hospital care upon production of their vaccination card, as long as the family has followed the appropriate referral route. In Tanzania **r**elatively low population density in some regions is perhaps one reason for a greater focus on providing inpatient care in health centres. In Tanzania, private health facilities are a smaller share of the system than in Kenya (26%) [17]; for-profit private facilities are primarily in the largest urban areas, while faith-based facilities are more geographically dispersed.

Even where hospital costs are covered by insurance or provided at nominal cost, patients often incur substantial out-of-pocket costs from hospital referrals. If publicly subsidized ambulance services are not available at the right time to transport referred patients to the next level of care (this is more of an issue in sub-Saharan Africa), families bear these costs. In some cases, a caregiver may stay free in the hospital with the child but may have to pay for his/her meals. If required diagnostic tests or medicines are not available in the facility, families may have to obtain these elsewhere at their own cost. These costs can deter adherence to hospital referral recommendations, especially where distances are large, where families have other children at home needing care, and where lost income is a factor.

## Materials and methods

The perspective adopted for economic analysis was that of the health sector, so as to assist country-level policy decision making. Although out-of-pocket costs are not part of this perspective, additional data were collected from the health sector about the magnitude of out-of-pocket costs associated with hospitalization, to assist in interpretation.

Ethical approval was obtained from the King George’s Medical University Internal Ethics Committee Ref ECR/262/Inst/UP/2013/RR-16, the Indian Council of Medical Research Ref 2020–9753, the Kenyatta National Hospital Ethic Review Committee Ref P333/06/2020 and KNH/ERC/R/235, the Comité National d’Ethique pour la Recherche en Santé ref SEN20/50, the Ifakara Health Institute Institutional Review Board Ref IHI/IRB/46-2020, the Tanzania National Institute for Medical Research Ref NIMR/HQ/R.8a/VolIX/3583, NIMR/HQ/R.8b/Vol. I/993 and NIMR/HQ/8.b/Vol.1/1183, and the WHO Ethics Review Committee Ref ERC.0003405, v2.4, 2 February 2023 and Ref ERC.0003406, v2.4, 19 November 2020. ERC.0003405 and ERC.0003406 document amendments to the original protocol, including lengthening the period of data collection (primarily due to COVID-19 pandemic), removing the CDSA arm in India, and incorporating small procedural changes informed by the three months of pre-testing in each country.

As described in more detail in [7], written informed consent was obtained from caregivers; in the case of an illiterate caregiver consent was documented by the signature of an impartial witness (India, Kenya, Tanzania) or the caregiver’s thumbprint and the signature of an impartial witness (Senegal). Informed written consent was also obtained for information from healthcare providers. Additional information regarding the ethical, cultural, and scientific considerations specific to inclusivity in global research is included in the Supporting Information (S1 Checklist: Inclusivity in Global Research).

### Data sources

Table 1 summarizes the contextual factors in each country. For example, there were variations across countries in the criteria for use of PO as well as the oxygenation level which was an indication for hospital referral. Start and end dates for collection of data used for the economic study are provided in Table 1.

**Table 1:** Contextual factors and sample sizes for cost calculations.

	India	Kenya	Senegal	Tanzania
Protocol for testing with PO	All sick children < 2 years		All infants < 2 months; all those 2–59 months with cough/difficulty breathing; all those 2–59 months with IMCI/CDSA class moderate (yellow) or severe (red)	
Criteria for referral	SpO2 < 90% Reinforced if SpO2 < 94% + severe illness	SpO2 < 90%	SpO2 < 92% Reinforced if SpO2 92 to <95% + severe illness	SpO2 < 90%
Location included	Rural only	Urban and rural areas		
Facilities included	PHC and CHC	Dispensaries & health centres	Postes de santé (health posts)	Dispensaries & health centres
Numbers of facilities (by type)	117 (74 PHC, 43 CHC))	60 (35 disp, 25 HC)	60 (all health posts)	66 (51 disp, 15 HC: in intervention 34 disp, 11 HC)
Dates for HC and hospital cost data collection	19 May 2023- 23 May 2023	2 Dec 2021 – 20 Feb 2022	Nov 22 2022 – Nov 25 2022	6 Sept 2022–15 Sept 2022
# of primary care (PC) facilities for economic data collection^1^	10 (6 CHC, 4 PHC: all rural)	9 (4 CHC, 5 dispensaries: 4 urban/peri-urban, 5 rural)	8 (all postes de santé: 4 urban, 4 rural)	18 (6 CHC, 12 dispensaries: 8 urban, 10 rural)
# of personnel (# medically qualified) at PC surveyed – range	5–54 (1–9: of which 1–6 qualified in Western medicine)	3–31 (2–12)	7–20 (2–7)	3–77 (2–44)
# of hospitals for economic data collection	N/A^2^	6	3	5
Dates for sick child observation data collection	N/A^2^	29 Oct 2021 – 5 Dec 2022	14 July 2021 – 12 Dec 2022	11 April 2021 – 17 Mar 2023

^1^CHCs and health centres (HCs) are facilities with a broader range of services; dispensaries (disp)/postes de santé/PHC’s have fewer services. In sub-Saharan Africa, the more basic primary care facilities are often headed by nurses and those offering more advanced treatment by clinical or medical officers. In India both PHCs and CHCs are headed by medical officers (or occasionally specialist doctors).

^2^Not available/not applicable: child observation data were also collected in India, but not available to authors at time of writing.

Economic data collection for the cost study was overseen by economists within each country team. These economists collected data from a number of those primary care facilities covered by the detailed TIMCI Service Provision Assessment surveys and stratified by level of care available (not relevant in Senegal), urban/rural location (not relevant in India) and size. Questionnaires were developed by the economists on the study team building on a template from the lead author, tailored to local conditions and administered using ODK (www.getodk.org) to the most senior medically qualified staff member at a given facility (medical officer-in-charge or chief nurse, as appropriate). The economists also sourced salary scale data at regional or national level. Data on prices of medical consumables were collected from the relevant national supply organizations.

The economists also collected data at those public facilities, usually hospitals, to which severely ill children are referred, from an appropriate resource person (senior medical officer or administrative officer). These data included selected information on charges for different components including costs of inpatient care, costs of ambulatory care (a fee for being assessed for intake, for those bypassing normal referral channels), cost of oxygen, ventilators, intensive care and meals for caregivers. Costs for travel from the primary care facility to the referral facility were obtained at the primary care facility. Costs incurred by caregivers and sick children in public hospitals do not generally cover the full economic costs, and the data collected were used primarily to assess financial barriers to care seeking. A bottom-up accounting of hospitalization costs for severely ill children was not feasible within the scope of the TIMCI cost study and costs were derived instead from a survey of the peer-reviewed literature. Since costing studies do not typically estimate cost of severe illness because of the heterogeneous conditions involved, severe pneumonia was used as the indicator condition.

Basic socio-demographic, clinical and outcome data were collected for all sick children 0–59 months of age attending study facilities for an initial consultation, with more in-depth studies of time flow (for patients) and observations of sick child consultations in a random stratified subset of the RCT [8] and pre-post facilities [9]. Children less than one day old, or attending for trauma, or routine care or for repeat visits within 28 days of prior enrolment were not eligible for inclusion [7]. In total, 50,580 children were included from the pre-post studies, and 157,677 within the RCTs. Cost-effectiveness was defined as cost per severe complication (as defined in the Introduction) averted. Interviews with caregivers, both at the time of visit, on Day 7 (by phone in India, Kenya and Senegal; in-person in Tanzania), and Day 28 (RCT only) were used, along with clinical records for enrolled sick children, to determine severe complications.

Involvement of Ministries of Health, civil society organizations and community advisory boards was sought in designing the research objectives, key outcomes and instruments and they are key partners in dissemination of findings. Engagement with local communities through civil society organizations and community health initiatives was used to reinforce the importance of attending health facilities to seek care. Ongoing studies present the qualitative findings from interviews of parents and healthcare providers, as well as key informant interviews of stakeholders subnationally, nationally and internationally, involved in policy, implementation or research in child health.

### Cost framework

The economic analysis assumed that change in costs with the intervention at primary facility level equalled:

Additional costs of equipment and training (amortized over a 3-year period)
+ Change in treatment costs (largely due to change in prescription behavior for antibiotics)
+ Change in costs of other diagnostic tests
+ Change in personnel costs (if introduction of PO and PO with CDSA changes consultation lengths)
+ Change in hospitalization costs (due to change in volume of hospitalizations if referrals change)
+ Recurrent cost of deployment and operation of CDSA algorithm in each country

A time horizon of one year was used for costs, hence no discounting, coinciding with the duration of the intervention. Training and equipment investments were amortized over three years using straight-line depreciation (no discounting) for simplicity. Some other smaller costs were not included, such as the cost of supportive supervision for the intervention: this was integrated into existing supervision and was difficult to cost. Each country incurred a one-time cost for development and deployment of the CDSA algorithm estimated as $80,400, which is not included, while recurrent costs were included (S1 Table). The one-time costs of CDSA would become a negligible amount per eligible child if scaled-up nation-wide and used for an extended period such as a decade, and there are likely also economies of scale in the recurrent costs.

### Details of cost calculations

Table 2 provides sources and details of the calculation of components of cost differences with the intervention, using WHO guidelines [18] and data from individual countries [14,19–22]. Increased cost of equipment and training (prices and quantities) were sourced from PATH country data. Diagnostic cost changes/differences (other than malaria tests, which were unchanged) were based on sick child observation data. Changes were noted for Tanzania, particularly with use of CDSA and in health centres (dispensaries do not always have diagnostics laboratories). Changes in treatment costs were based on observed use of amoxicillin in sick child observation data. Prescription of amoxicillin dominated use of all other antibiotics, and no significant change/difference in use of other antibiotics was observed (authors’ calculations). Decreases in antibiotic usage would potentially have benefits of reduced future costs of antimicrobial resistance but measuring this was beyond the scope of the study. Personnel cost changes/differences utilized change/difference in consultation lengths from time-flow data. Salary data were collected either at the facility level (contract personnel), or by collecting information on civil service employee’s grade/cadre level at the facility level, combined with centrally-collected information on public sector employees’ salaries and benefit entitlements (see Supporting Information S1 File for further details). Costs are presented in US dollars of 2021 (2022 for India), using the following exchange rates from OANDA (https://www.oanda.com): 1 Indian rupee = $0.0127 USD; 1 Kenyan shilling = $0.0089 USD; 1 West Africa CFA franc = $0.017 USD; 1 Tanzanian shilling = $0.00043 USD.

**Table 2:** Cost and other parameters required to estimate effect of intervention.

Line No.	Cost Item	India (2022 $)	Kenya (2021 $)	Senegal (2021 $)	Tanzania (2021 $)
**Unit costs**					
1	Additional training cost per year per facility^1^	$26.55 (PHC & CHC)	$330.7 (disp + HC)	$347 (poste de santé)	$184.1 (disp) $368.2 (HC)
2	Additional equipment cost per year per facility^2^	$83 (PHC) $167 (CHC)	$132.40 (disp) $264.80 (HC)	$95.30 (poste de santé)	$95.30 (disp with PO only); $132.40 (disp PO & CDSA), double for HC
3	Median cost per child per course of amoxycillin^3^	$0.33	$0.35	$0.44	$0.33
4	Health sector cost per hospital stay for child w’ severe pneumonia^4^	$101.53	$141.70–$165.17	$180.03	$106.70
5	CDSA recurrent annual cost per facility^5^	n/a	$267		
**Quantities**					
6	Intervention effect on antibiotic use^6^	Control 74.3% (17,842/24065), PO 68.4% (17085/23384))	↓ use from 83.6% (7917/9469) to 54.6% (11442/20975)	↓ use from 62.2% (5162/8292) to 41.0% (4859/11844)	Control 67.7% (22628/33441) PO 67.7% (23799/35166), CDSA 62.1% (24343/39191)
7	Intervention effect on use of diagnostics other than malaria^7^	Data not available	very few non-malaria tests occur	very few non-malaria tests occur	no major difference in PO arm; modestly higher in PO+CDSA arm
8	Intervention effect on hospitalization^8^	+0.1% (PO 27/24966; control 3/24065)	No change (post 6/20975; pre 4/9479)	-0.1% (post 7/11844; pre 9/8292)	+0.1% (PO 32/36014; control 17/33441) +0.1% (PO+CDSA 57/39191)
10	Average number of sick children seen per week per facility^9^	21.0 (PHC) n = 4 58.3 (CHC) n = 6	47.8 (disp) n = 34 79.6 (HC) n = 22	10.6 (postes de santé) n = 58	42.0 (disp) n = 48 111.2 (HC) n = 7
11.	% of sick children visiting, eligible for study participation^10^	78.5% (49031/62423)	95.6% (30444/31843)	94.2 (20136/21372)	98.0% 106526/108646
12	% of those eligible for study particip., tested with PO^11^	94.5% (23591/24966)	82.8% (17360/20975)	44.2% (3362/7607)	45.8% (25694/56092)

^1^**All countries:** assume roll-out training amortized over 3 years, using straight-line depreciation. Costs include per diems for resource person and participants per government policy, cost of travel (where required), cost of training facility and refreshments. Costs exclude research project-related costs and cost of training of trainers. **India:** 5 one-hour sessions online IMNCI (Integrated Management of Neonatal and Child Illness) refresher; one-day on-site training for PO; CDSA not included in roll-out. Format did not require per diems. **Kenya:** 7 days training (IMCI refresher, PO, CDSA), all in-person, 1 person trained per facility, plus print materials. **Senegal:** 6 days training (IMCI refresher, PO, CDSA), all in-person, 2 people trained per facility. **Tanzania**: 5 days training (IMCI refresher, PO, and where relevant CDSA), all in-person, plus printed IMCI materials. 1 person trained per dispensary, 2 per health centre.

^2^**All countries:** Cost of PO was $250 for India ($286 including shipping in Africa); cost of tablet for CDSA was $171.30 (source: PATH procurement records). Typical allocation was that PHC/postes de santé/dispensaries received one PO and one tablet, CHC/Health centres two of each (where CDSA was implemented). Assume equipment amortized over 3 years using straight line depreciation. Maintenance of CDSA required approx. 20 mins/week online from an IT officer covering around 40 health facilities according to PATH country representative – no cost included as was not possible to get a salary estimate.

^3^**All countries:** uses WHO’s recommended dose 40mg/Kg, twice per day for three days [18]; and WHO growth charts (averaged for boys and girls) for mean weight of child 30 months of 13.05 Kg, i.e., cost for 15 250 mg dispersible tablets (sold in package of 100). **India** http://www.gem.gov.in; **Kenya** PATH procurement from Kenya Medical Supplies Authority; **Senegal** PATH procurement from UNICEF Supply Division; **Tanzania** PATH procurement from Medical Stores Department.

^4^Authors’ calculations: **India**: source [19]: **Kenya**: lower estimate source [14]: higher estimate source [20]: Senegal: source [21]: **Tanzania:** modeled, see Supporting Information S1 File for details.

^5^Annual cost of maintaining CDSA (country average): estimates from co-authors involved in deployment. See Supporting Information S1 Table for details.

^6^Proportion of children prescribed an antibiotic on same day as consultation (only systemic antibiotics included): source [8,9].

^7^Authors’ calculations using sick child observation data. Cost per single PoC urine dipstick and hemoglobin strip: source [22]. Other tests used included stool tests, HIV, urinalysis and full blood. Cost data for stool tests obtained by PATH from local councils; assume “other” laboratory tests cost same as stool tests. No significant change in malaria test numbers in Kenya, Senegal and Tanzania (data not available for India).

^8^Proportion of children completing referral to hospital by Day 7: source [8,9]

^9^India: means for 4 Primary Health Centres (PHCs) and 6 Community Health Centres (CHCs) from facilities in economic data sample, using facility records, Jan-Mar 2023; African countries: means from facilities initially considered for inclusion in study (these largely overlap with those ultimately included), using facility records Jan-Oct 2021.

^10^Out of all sick children in the 0–5 age group visiting the facility, those ineligible included those less than 1 day old, trauma patients, and those entering inpatient care [7]. Assumes that rate does not differ between more basic facilities (PS/disp/PHC) and more advanced facilities (HC/CHC).

^11^Source [8,9]

Literature searches were undertaken for hospitalization costs for severe pneumonia in children using published studies. Costs were updated to 2021 using standard methods: cost in local currency of study year were updated to 2021 (Africa) or 2022 (India) using the domestic consumer price index from IMF (https://data.imf.com) and converted to USD of 2021 (Africa) or 2022 (India) using the market exchange rate obtained from OANDA (https://www.oanda.com). For Tanzania, where no relevant hospitalization cost could be identified from the literature, and for which insurance charge data could not readily be obtained, the proxy used was based on 1.35 times the estimated full bed-night costs in Tanzania (using Madsen et al’s results) [19] for a stay of six days (based on median from hospitals surveyed). Bed-night costs also had to be estimated since the bed-night charges to patients in Tanzania (ranging from $1.29 to $4.30 across the three referral hospitals for which data could be obtained) are very nominal and do not cover salary costs. See Supporting Information S1 File for further details of the methods for hospitalization cost estimates for Tanzania.

## Results

Table 2 summarizes the difference in quantity of cost items with the intervention. Some costs (e.g., equipment and training, operation of CDSA) are determined according to the facility level, while others depend on numbers of children tested (e.g., antibiotic prescription). Average numbers of sick children seen per facility and proportions of children eligible for testing and actually tested (Table 2) were used to calculate standardized costs per 100 children tested (S2 Table). While India and Kenya stated that all sick children in the study should be tested with PO, the criteria in Senegal and Tanzania were more restrictive (Table 1). In India a considerably larger proportion of sick children were suffering from injuries (trauma) and hence not eligible for testing. Primary health centres and community health centres in India are expected to have X-ray facilities whereas primary care facilities in Kenya, Senegal and Tanzania generally do not, likely causing the difference in use patterns.

No change in personnel cost is included. Preliminary analysis of the time-flow data suggested that there were differences in consultation lengths across the different study arms (countries using RCT) and comparing before and after intervention (pre-post studies). However, analysis of data from the self-reported time-use survey conducted by the economists suggested that time reported to be available by qualified staff for sick child consultations, combined with information on the number of sick child consultations per week from facilities’ records data (Table 2) was ample to allow for any increases in consultation length observed in the time flow studies. Other studies of time flow of health care providers suggest that although providers are very busy at some times, there are other times when they may be waiting for patients [23]. Of course, this does not take into account that at very busy times, wait times for patients might increase with the intervention.

In two countries (Kenya and Senegal) there were reductions in antibiotic use with the intervention of almost 30 percentage points in Kenya and just over 20 percentage points in Senegal. This is likely related to use of the CDSA. In Tanzania there was a smaller reduction in the arm using CDSA (5.6 percentage points) and in India in the arm using PO (5.9 percentage points). Antibiotic prescription rates prior to the intervention ranged across the four countries from 62.2% to 83.6% of sick children presenting at the facilities prior to the intervention, considerably above the 30% ceiling for those presenting at primary health facilities seen by WHO as reasonable [24].

There were small changes in use of diagnostic tests other than for malaria, with minor effects on cost. In all three African countries, malaria tests are used extensively (on almost 50% of sick children in Tanzania, 50–60% in Senegal with seasonal variation, and around 20% in Kenya: TIMCI records), but no significant change in malaria testing was observed with the intervention. In Tanzania, however, use of additional diagnostic tests along with a malaria test was slightly higher in the CDSA arm (11.5% of children: 94/818) as compared to the PO arm (7.5%: 56/744) and the control arm (2.9%: 25/851). This difference was larger for health centres which have laboratories, compared to dispensaries which do not always have them. The most commonly added tests were urinalysis, stool and hemoglobin tests. This may be due to prompts from the CDSA to the healthcare provider, depending on patient data that is entered.

Adherence to recommendations for referral to hospital and hence hospitalization costs changed only marginally, being slightly higher with the intervention both India and Tanzania and slightly lower in Senegal with no observed difference in Kenya. Adherence here is defined as attendance at hospital either on the day of the consultation or the next day. Rates of adherence with recommendations for referral are low: only about a quarter of those referred in India and Senegal, and less than 10% in Kenya and Tanzania, comply [8,9].

**Fig 1:**
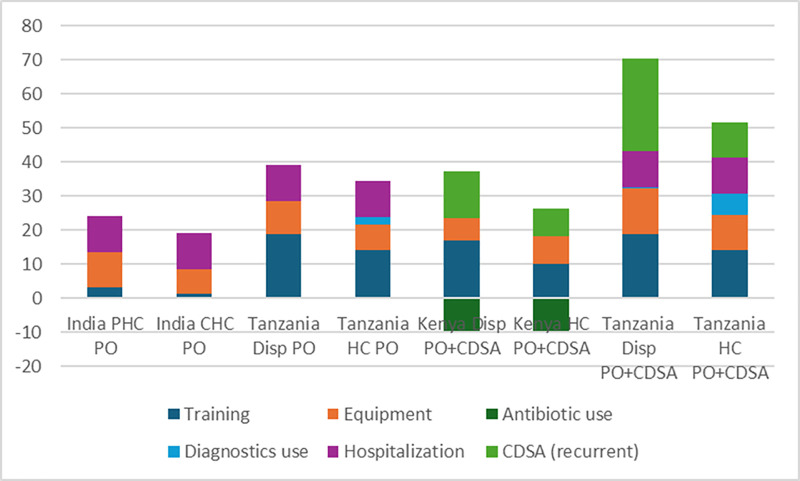
Cost component changes ($) with intervention, per 100 children tested, by facility: Cost component changes for Senegal for postes de santé using PO plus CDSA are: training + $151.30; equipment + $41.20; antibiotic use -$9.32; diagnostics use no change; hospitalization -$18.03; CDSA + $116.45. Details provided in S2 Table.

Fig 1 compares the effect of the intervention on the different cost components, both for PO only and PO plus CDSA, in both cases compared to standard care. Cost of additional equipment, training and operating the CDSA for the intervention are the largest items and are larger in the three sub-Saharan African countries than in India. This is primarily because India used five hours of online refresher training for IMCI, as compared to five to seven days in-person in the other countries. Factors increasing training costs include longer training duration, training modality (in-person versus online), size of mandated per diem for off-site in-person training, and whether one or two individuals per facility were trained. India also did not use the CDSA except for a short trial in a limited number of facilities. Recurrent costs of using the CDSA (both initial deployment, and annual operating costs) are substantial.

These costs were partially offset in those countries where antibiotic use dropped (Kenya and Senegal) or where hospitalization rates dropped (Senegal), while slightly higher use of diagnostic tests (other than malaria) increased net costs slightly for Tanzania.

**Fig 2:**
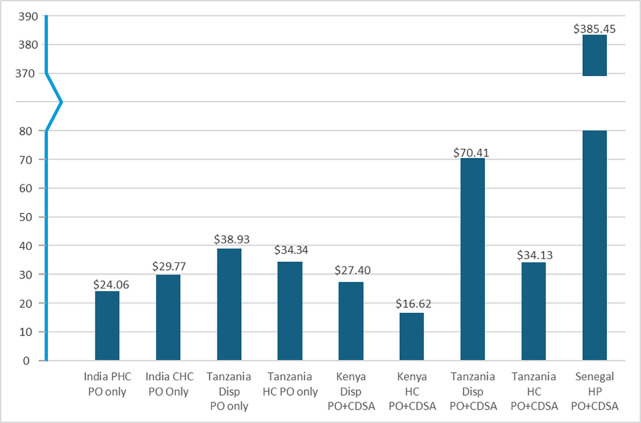
Net cost change ($) with intervention, per 100 children, by facility.

Net costs per hundred children tested (Fig 2) were lowest for Kenya at $16.62 at HCs using PO and CDSA, and highest in Tanzania at $70.41 at dispensaries using PO and CDSA. The net costs were an outlier in Senegal at $384.45 where the intervention was introduced only in the smaller basic primary care facilities, where numbers of eligible children attending the facility were a factor of ten smaller than in the busiest facilities (health centres in Tanzania). Net costs per child tested were lower in all countries at the more advanced primary care facilities (greater patient volumes) than the more basic facilities.

Given that the rate of severe complications was slightly higher in the RCT intervention arms [8], the conclusion is that usual care dominated the introduction of PO or PO plus CDSA, in this study. I.e. costs of the intervention were higher and the outcomes were slightly worse. Hence Incremental Cost Effectiveness Ratio was not calculated.

The economics survey results at both primary care and hospital level provide some context on the barriers to hospitalization (Table 3). Cost of care for under-five children with infectious diseases in public hospitals is very low in India, and nominally free in Kenya, Senegal and Tanzania for those referred from primary care. However, budget shortfalls in the health system can lead to stockouts at health facilities, requiring patients at times to have to pay for their own diagnostic tests and medications.

**Table 3:** Data on hospitalization costs, some of which may be borne by patient families, from economic surveys.

Data from survey of primary care facilities				
	India (n = 10)	Kenya (n = 9)	Senegal (n = 8)	Tanzania (n = 18)
Median distance to referral centre (range) in Km	35.5 (20–60)	17.5 (1–25)	6.5 (0.5–50)	10 (1–98)
Median cost of travel to hosp (range)^1^ Public transport	$1.02 ($0.25–$2.94)	$8.90 ($2.2–$20.2)	Taxi is used if no ambulance available, no public transport	Only one centre indicated public transport was an option
Same, private transport	$8.6 ($5.8–$31.5); $1.27 ($1.27-$12.27) if own car	$11.35 ($4.90–$17.80)	$5.15 ($1.71–$25.65) (taxi)	$3.60 ($0.96–$8.40)
**Data from survey of referral hospitals**				
	**India**	**Kenya (n = 6)**	**Senegal (n = 3)**	**Tanzania (n = 5)**
Facility types surveyed	N/A^2^	3 subcounty & 3 county hospitals	3 district hosps	1 regional hosp, 1 private hosp, 1 district hosp, 2 small hosps^3^
Median inpatient cost/day (range)	N/A	$2.00 (0–$4.45)	$3.40 (0–$17)	$2.15 (0–$6.45)
Median ambulatory cost^4^ (range)	N/A	$4.45 (0–$13.35)	$0	$4.30 ($2.15–15.05)
Median cost per day meals for caregiver (range)	N/A	$2.23 (0–$5.34) 1 of the 6 facilities doesn’t provide meals to caregiver	No meals provided for caregiver	Caregiver has to pay for own meals
**Average monthly earnings, employees, national surveys, all skill levels, USD [** 25 **])**				
	$239.20 (2022)	$136.6 (2019)	$146.7 (2019)	$186.7 (2020)

Data in this table were reported by the medically qualified individual in charge (at the primary care facility), and either the senior medical officer or senior administrator (at the hospital).

^1^Most facilities report that an ambulance paid by the health system can be obtained, if it is available, and costs are borne by the facility. However, note that the most sparsely populated district surveyed in Tanzania, four of the five facilities reported that no ambulance service existed. Costs are reported in US $ of 2021 (African countries) and 2022 (India)

^2^Not applicable

^3^Patients in one district of Tanzania are referred to health centres; costs for the two health centres in this district which were surveyed are not included in the table

^4^Patients not referred from a lower-tier facility must pay this cost to be assessed as outpatients prior to potential admission

Almost all the primary care facilities noted that ambulance transport for referrals was free of charge if available. The one exception is the least densely populated district surveyed in Tanzania (Kaliua) where no ambulance was available at four of the five health centres, and while it was available at the fifth centre, the referral hospital was 98 Km away. Frequently, however, in sub-Saharan Africa ambulances are not readily available. Median costs of public transport to hospital were $1.02 in India and $8.90 in Kenya, but not a common option in Senegal and Tanzania. Median ambulatory care fees were $4.45 in Kenya and $4.30 in Tanzania (i.e., fees for children arriving at hospital without referral). Kenya provides meals to caregivers accompanying children at a median cost of $2.89/day, but these are not provided in Senegal and Tanzania.

## Discussion

The results have various implications. They point out the importance of understanding costs to patients which affect health-seeking behavior as well as response to recommendations to refer to hospital. They provide suggestions as to how to reduce implementation costs, both in terms of methodologies for training and supervising of healthcare providers, and in moving to integrated digital healthcare solutions rather than stand-alone interventions.

The IMCI guidelines place considerable emphasis on identifying severe disease and appropriately referring severely ill children. Use of PO and CDSA can assist in identifying severe disease. However, if families find it difficult to comply in a timely manner with referral recommendations, this reduces potential health benefits. Direct costs borne by households (costs of caregiver meals, cost of ambulatory care and cost of taking taxis to hospital) may seem low. However, these costs are significant if compared to average daily earnings in the four countries of $11.07 (India), $6.32 (Kenya), $6.79 (Senegal) and $8.64 (Tanzania). (This calculation used average monthly earnings, for the latest year available [25], assuming 260 workdays per year and 21.6 workdays per month on average). Other costs may also face families at hospital, if diagnostics and treatments are not available or not provided free of charge, or if unofficial payments are expected to obtain care. Indirect costs to household include foregone income, and costs of arrangements made for other children at home, such that a caregiver can accompany a child to hospital. These factors may explain the low adherence rate to referral recommendations noted above [8,9]. In turn, this may help explain why unexpected results were obtained for severe complications, where the severe complications were composed of delayed hospitalization, hospitalization without referral and death. Other considerations as to why the severe complications did not move in the expected direction with the intervention are discussed elsewhere [8,9].

The trial could not control for potential changes in health seeking behavior with the intervention. Families do seek out facilities known to have better availability of treatments at a given time [26,27]. Calculations using the most recent Demographic Health Survey data from each country (https://www.statcompiler.com/en/) suggest that only a minority of those with children with acute respiratory infection seek care at a public primary care facility (5.7% in India; 34.6% in Kenya, 40.6% in Senegal and 40.0% in Tanzania). In order to remove biases in intervention findings, the implementing agency (PATH) ensured that necessary key basic supplies were consistently available in the study facilities; however, this could have affected health seeking behavior.

The empirical results suggest that the previous modelling studies were too optimistic in cost and cost-effectiveness estimates [5,10,11]. They did not allow for non-compliance with recommendations for referral, and two of the three studies [5,11] did not include costs of refresher IMCI training, without which use of PO is likely to be compromised and did not predict the reduction in inappropriate antibiotic usage which was observed here.

The results suggest ways in which the intervention can be implemented at lower unit cost. One possibility is doing some of the training online. Other studies have examined the effectiveness of hybrid and online training for IMCI at lower cost but without losing effectiveness [28]. Focusing the introduction of PO (with or without CDSA) in facilities with greater volumes of sick child visits is another possibility. Increasing the proportion of sick children actually tested compared to those eligible for testing is a third possibility. Two interventions employed in Kenya may be helpful for improving testing rates. First, Kenya emphasized innovations in supportive supervision, such as using WhatsApp groups for device users in different facilities. A systematic review of this topic in sub-Saharan Africa notes that it improves job satisfaction and health worker motivation [29], but there is insufficient evidence as to impact on clinical outcomes. Second, Kenya is the only country (other than a limited number of observations in Tanzania) where PO was deployed in about half the use cases in a triage step, and the other half during the consultation itself (data from time-flow study).

The experience with CDSA costs highlights the difficulty of introducing a digital application as stand-alone, where all the costs of servers, facility-level devices (tablets), IT support and management etc. are borne by a single intervention with limited coverage. This component of the intervention would be less costly if integrated into a systems intervention and used at large scale.

Strengths of the study for economic analysis include the large samples and inclusion of four countries and a variety of contexts, as well as the rich data sets with a wealth of information. Limitations imposed by budget constraints include the relatively small size of the time flow and sick child observation datasets used to calculate some of the input costs, the use of a health sector rather than a societal perspective and the use of existing hospital cost studies rather than a *de novo* bottom-up accounting of hospital costs. Reliance on expert opinion to adapt the actual costs in a research study, to likely costs in a scaled-up intervention, is also a limitation.

## Conclusions

The cost and cost-effectiveness results illustrate the difficulty of introducing a technological solution in an under-funded health system and the importance of considering behavior change, a lesson that proved important in the introduction of malaria rapid diagnostic tests [30]. Large-scale clinical trials such as the TIMCI one are extremely useful prior to broader roll-out of new interventions. Although the COVID-19 pandemic underscored the important of using PO in assessing severe respiratory illness, the costs involved may make this difficult to extend to sick children visiting smaller primary care facilities in LMICs.

## Supporting information

S1 File PubMed search details and additional cost details. (DOCX)

S1 Table Cost of development and deployment of CDSA algorithm (average for one country, based on three-country experience). (DOCX)

S2 Table Increase (+) or decrease (-) in costs per 100 children tested per year using PO or PO and CDSA. (DOCX)

S1 Checklist Inclusivity in Global Research: TIMCI. (DOCX)
