# Supplementary material for: Costs of testing sick children in primary care with pulse oximetry: Evidence from four countries, both with and without electronic clinical decision support
Source: PLOS Glob Public Health. 2025 Jul 1;5(7):e0004644. doi: 10.1371/journal.pgph.0004644 (PMC12212478; doi:10.1371/journal.pgph.0004644)
Supplement: S1 File — (DOCX) [file pgph.0004644.s001.docx]

**S1. Supporting Information: Costs and cost-effectiveness of testing sick children in primary care with pulse oximetry.**

**PubMed search details**

The authors searched PubMed on May 2, 2024, using the search terms (pulse oximet*[Title/Abstract]) AND (cost[Title/Abstract]), and ((pulse oximet*[Title/Abstract]) AND (cost-effectiveness[Title/Abstract])) with no restrictions on date or language. Inclusion criteria were:

1. Studies were at primary care level;
2. Studies contained information on children under five;
3. Studies focused on infectious disease;
4. Studies focused on low- and middle-income countries;
5. Effectiveness outcomes considered included a health measure.

**Additional cost details**

1. **Training cost**

National level training-of-trainers was excluded; costs of attendance of PATH employees at training sessions was excluded (aim was to make the best estimate of cost of roll-out in practice, rather than cost of a pilot roll-out intervention with strong research orientation). Costs included government-mandated per diems for trainees and trainers ($0 in India for trainees; $100–$110 in Kenya; $25 in Senegal; $55 in Tanzania); cost of training venue rental with refreshments where relevant; cost of printed materials, cost of transport of participants where relevant.

1. **Imputed hospitalization cost, Tanzania**

We used a study from India which identified that 74% of the hospitalization costs for severe pneumonia were the bed-night costs, for a non-profit second-level hospital [1]. This 74% assumption was tested against actual hospital stay cost data for pneumonia for children for Kenya [2] and yielded an estimate 16.7% higher than the actual reported hospital stay cost, which was considered reasonable. For Tanzania, bed-night charges to patients are very nominal (reported as $2.15 per night: table 4); however this does not cover the cost of health worker salaries. One alternative is to use the bed-night cost in non-profit hospitals (but allowing for a 14% markup in the non-profit sector over public facilities). Expert opinion (Dr. Kaushik Ramaiya, CEO Shri Hindu Mandal Hospital in Dar-es-Salaam) suggested that non-profit hospitals in Tanzania charge $15–$45 per bed-night, with the variation corresponding to the type of services provided. If we use the lowest figure and remove the 14% overhead, that gives an estimated bed-night cost of $13.16 for Tanzania. The cost for six nights, multiplied by 1/0.74, gives an estimated cost per hospital stay for severe pneumonia of $106.70.

1. **Personnel costs**

The economics surveys included data on reported time allocation of those personnel qualified to consult with sick children, to this activity. Time allocated to other activities was also collected, namely time spent in routine consultation with children under 5; time spent in consultation with others (children 5 and over; adults); time spent with supervisor; time spent supervising other staff; time spent in administrative tasks; time spent in other tasks as relevant by country. Response choices were less than 0.5 days per week, 0.5–1 days per week; 1–2 days per week, 2–3 days per week, 3–4 days per week, 4–5 days per week, more than 5 days per week, other (specify)

Information necessary to estimate individuals’ salaries was also collected, appropriate to country context (Job cadre, scale where relevant, and allowances received in Kenya; grade, monthly base salary and list of allowances to which entitled; Job cadre and salary grade in Tanzania; in India only medical doctors are permitted to conduct consultations with sick children. Some salary information could be collected at facility level (salary of contract employees, non-civil service employees, and all employees in Senegal). In other countries centralized sources were consulted.

For India, data on medical officer salaries and allowances were obtained from the district health society office records. For Kenya: Salary scales for 2021 were used from one of the three counties, and the mid-point of the salary range for each specific cadre was used (not available online but are similar to published salary scales [3] and published allowance scales [4]. For Tanzania: civil service salary data are not publicly available. We used instead data from a detailed survey of 704 medical professionals, conducted in 2012 for one district of Morogoro region, to obtain estimates of median salary of relevant medical professionals employed at primary facilities (pers. Comm. Dr. Abdallah Mkopi, Ifakara Health Institute). This was supplemented/cross-checked against information from the TIMCI economics survey which obtained salary information for healthcare providers working currently in the health facilities but paid by another agency (often an NGO) as support to the Tanzanian healthcare system, for staff categories which did not appear in the 2012 survey. We did not update the salary data from 2012 since civil service salaries in both Kenya and Tanzania are often not updated for several years.

1. **Cost of developing, deploying and operating the electronic CDSA algorithm.**

Developing and deploying the CDSA algorithm in a specific country required a series of steps. Country-specific guidelines were obtained and discussed with the country. The medAL software ( <https://medal-suite.com>) developed by Unisanté was used to translate these guidelines into a programmed form. The algorithm was then updated following both desk-based and field testing. Once finalized, the software was installed on the tablets for distribution to individual health facilities. Costs of training healthcare providers were included in the training costs and costs of the tablets included under equipment (described above). There are recurrent costs of providing ongoing support to maintain the software, host data, etc.

S1 Table provides estimated costs per country (averaged across the experience with the three different countries using CDSA throughout the trial). These costs could likely decrease if a new country wished to adopt a similar algorithm, in that some of the work initially done by the team at Unisanté could be devolved to country level (cost per appropriately qualified person-day in Switzerland are estimated as $800, compared to $200 in-country). The onetime costs are unlikely to vary too much by number of facilities in-country using the algorithm and are estimated as $80,400 per country (based on the mid-point of the estimates of the sum of column 1 and column 2).

However, the recurrent costs (costs of deployment and operation) vary according to number of health facilities covered. We assume 60 facilities per country are covered (this is similar to the number in Kenya and Senegal, where all facilities used the CDSA: in Tanzania in fact only one-third actually used the CDSA but we maintained the same cost per facility as in Kenya and Senegal for consistency).

**References**

1. Madsen HO, Hanehoj M, Das AR, Moses PD, Rose W, Puliyel M, et al. Costing of severe pneumonia in hospitalized infants and children aged 2–36 months, at a secondary and tertiary level hospital of a not-for-profit organization. Trop Med Int Health*.*2009; 14: 1315–22. <https://doi.org/10.1111/j.1365-31>

2. Ayieko P, Akumu AO, Griffiths UK, English M. The economic burden of inpatient paediatric care in Kenya: household and provider costs for treatment of pneumonia, malaria and meningitis. Cost Effec Resource Alloc. 2009; 7: 3. <https://doi.org/10.1186/1478-7547-7-3>

3. Salaries and Remuneration Commission, Kenya. Phase IV salary review for county government employees at the executive. Effective 1^st^ July, 2020. <https://src.go.ke/wp-content/uploads/2020/07/Phase-IV-Salary-Review-for-County-Government-Employees-at-the-Executive.pdf>. Accessed August 9, 2022.

4. Salaries and Remuneration Commission, Kenya. Health Workers Allowances. Ref. no. SRC/TS/CGOVT/3/61/ Vol III/ (136) 14 September 2015. [https://src.go.ke/wp-content/uploads/2015/09/Health-Circular.pdf. Accessed July 21, 2022.](https://src.go.ke/wp-content/uploads/2015/09/Health-Circular.pdf.%20%20Accessed%20July%2021,%202022.%20%20%20)
