# Supplementary material for: Costs of testing sick children in primary care with pulse oximetry: Evidence from four countries, both with and without electronic clinical decision support
Source: PLOS Glob Public Health. 2025 Jul 1;5(7):e0004644. doi: 10.1371/journal.pgph.0004644 (PMC12212478; doi:10.1371/journal.pgph.0004644)
Supplement: S1 Table — (DOCX) [file pgph.0004644.s002.docx]

**Table S1. Cost of development and deployment of CDSA algorithm (average for one country, based on three-country experience)**

| **Cost item** | **Onetime costs –**  **International** | **Onetime costs - local** | **Recurrent costs - local** |
| --- | --- | --- | --- |
| Gathering guidelines / defining scope of adaptation; inc. desk review and workshops | 5-10 | 5-20 |  |
| Drafting country-specific human-readable algorithm | 10-20 | 5-15 |  |
| Programming | 10-20 | 10-20 |  |
| Desk-based testing | 5-10 | 5-10 |  |
| Piloting | 5-20 | 15-40 |  |
| Updates following piloting (algorithms, programming, testing) | 5-20 | 10-20 |  |
| Set-up and configure software(s) | 5 |  | 2 |
| Software maintenance corrective and security |  |  | 12 |
| Clinical updates |  |  | 2-10 |
| Data management |  |  | 12 |
| Tablet initial configuration and deployment (assume once per 3 years) |  |  | 10 (once per 3 yrs, i.e. 3 days per year) |
| User Support |  |  | 10 - 30 |
| IT coordination / management |  |  | 12 |
| Server configuration | 2 | 2 |  |
| TOTAL PERSON-DAYS FOR ONE COUNTRY | 47 - 107 | 52 - 127 | 53-71 person days |
| TOTAL COST OF PERSON-DAYS | $37,365 – 85,065 | $10,400 – 26,800 | $10,600 – 14,200 |
| DATA HOSTING |  |  | $3,600 |
| TOTAL ANNUAL COST INCLUDING HOSTING |  |  | $14,200-$17,800 |
| TOTAL ANNUAL COST PER FACILITY |  |  | $267 |

Excludes costs for capital equipment replacement (e.g. servers, routers); cost of tablets is included elsewhere in equipment costs. Excludes costs of integration, governance and other factors found in the Digital Square Total Cost of Ownership Tool (TCO) - <https://digitalsquare.org/tco-tool>. Costing assumes that the CDSA is not interoperable, that training on use of the CDSA is included in the broader intervention package and that governance costs are not needed.
