# Supplementary material for: Costs of testing sick children in primary care with pulse oximetry: Evidence from four countries, both with and without electronic clinical decision support
Source: PLOS Glob Public Health. 2025 Jul 1;5(7):e0004644. doi: 10.1371/journal.pgph.0004644 (PMC12212478; doi:10.1371/journal.pgph.0004644)
Supplement: S2 Table — (DOCX) [file pgph.0004644.s003.docx]

**Table S2. Increase (+) or decrease (-) in costs per 100 children tested per year using PO or PO and CDSA**

|  | **India (2022 $)** | **Kenya (2021 $)** | **Senegal (2021 $)** | **Tanzania (2021 $)** |
| --- | --- | --- | --- | --- |
| Training cost  PHC/PS/disp +PO  Same, + PO & CDSA  CHC/HC + PO  Same, + PO & CDSA | +$3.28  n/a  +$1.18  n/a | n/a  +$16.81  n/a  +$10.09 | n/a  +$151.20  n/a  n/a | +$18.78  +$18.78  +$14.19  +$14.19 |
| Equipment cost/year/’00 children tested  PHC/PS/disp +PO  Same, + PO & CDSA  CHC/HC + PO  Same, + PO & CDSA | +$10.25  n/a  +$7.43  n/a | n/a  +$6.72  n/a  +$8.08 | n/a  +$41.52  n/a  n/a | +$9.72  +$13.51  +$7.34  +$10.21 |
| Cost savings in antibiotic | $0 | -$9.70 | -$9.32 | $0 |
| Cost increase in diagnostics  PHC/PS/disp +PO  Same, + PO & CDSA  CHC/HC + PO  Same, + PO & CDSA | Not available | No clinically significant dfference | No clinically significant difference | -$0.24  +$0.21  +$2.14  +$6.22 |
| Hospitalization cost Increase or decrease | +$10.53 | $0 | -$18.03 | +$10.67 |
| CDSA operating cost/  PS/disp PO+CDSA  HC, PO & CDSA | n/a  n/a | +$13.40  +$8.15 | +$116.34  n/a | +$27.24  +$10.29 |
| Net cost change  PHC/PS/disp+PO  Same, + PO + CDSA  CHC/HC + PO  Same, + PO & CDSA | +$24.05  n/a  +$19.14  n/a | n/a  +$27.40  n/a  +$16.62 | n/a  +$385.45  n/a  n/a | +$38.93  +$70.51  +$34.34  +$34.13 |

Costs calculated from data in Table 2; n/a means not applicable
