# Supplementary material for: Costs of testing sick children in primary care with pulse oximetry: Evidence from four countries, both with and without electronic clinical decision support
Source: PLOS Glob Public Health. 2025 Jul 1;5(7):e0004644. doi: 10.1371/journal.pgph.0004644 (PMC12212478; doi:10.1371/journal.pgph.0004644)
Supplement: S1 Checklist — (DOCX) [file pgph.0004644.s004.docx]

**S1. Checklist: Inclusivity in global research**

**Ethical considerations, permits and authorship**

*This section is applicable to all research types.*

Provide details as to who granted permissions and/or consent for the study to take place in the Methods section of your manuscript. This should include the names of **all** ethics boards, governmental organizations, community leaders or other bodies that provided approval for the study. If individuals provided approval refer to these people by their role or title but do not list their name(s).

Reported in paragraph 2 in Materials and Methods (note that page numbers for the final manuscript are not available during the review process)

If there were any deviations from the study protocol after approval was obtained please provide details of these changes in the Methods section of your manuscript.
Did this study involve local collaborators that are residents of the country where the research was conducted or members of the community studied? If you do not have any authors from said communities, please provide an explanation for this below.

The 24 named authors of the study include 13 authors from the four countries where research was conducted (generally the PI, the PATH country lead and the country economist), and 11 authors from Swiss TPH/PATH USA/University of Waterloo. The larger TIMCI collaborator group <https://zenodo.org/communities/timci/about> includes an additional 29 individuals from the four countries, and an additional 26 individuals from the Swiss Tropical Research Institute and PATH (USA). The research was conducted in 303 health facilities, in 10 divisions in 1 state of India, 3 counties in Kenya, 5 districts in 1 region of Senegal, and 3 regions of Tanzania; incorporating collaborators from every single local community concerned who also met the PLOS’ criteria for authorship was not practicable.

Reported at end of paragraph 2 in Materials and Methods

Everyone listed as an author should meet PLOS’ criteria for authorship and all individuals who meet these criteria should be included in the author byline, rather than the acknowledgements. For further information please see the journal’s Authorship Policy.

**Human subjects research (e.g. health research, medical research, cross-cultural psychology)**

Did you obtain written informed consent from a representative of the local community or region before the research took place? How did you establish who speaks for the community? Details of written informed consent obtained from study participants should be reported separately in the Methods section of your manuscript.

The four country PIs, all co-authors (Drs. Shally Awasthi, Honorati Masanja, James Machoki M’Imunya and Ousmane Ndiaye) were responsible for obtaining the written informed consent from the appropriate communities and the appropriate health organization levels, as required by national ethics review bodies. They were supported in this by the four PATH country leads, who are also co-authors (Maymouna Ba, Andolo Miheso, Deusdedit Mjungu and Kovid Sharma). The process for obtaining written informed consent from study participants is detailed in paragraph 3 of the Methods section, which also references that additional details for this are available in the published Protocol.

How did members of the local community provide input on the aims of the research investigation, its methodology, and its anticipated outcome(s)?

Engagement with the many local communities involved is described in the published Study Protocol and the final paragraph of the Data Sources section and occurred via the relevant community health workers/volunteers (whose title/exact role varies by country), as well as through local civil society organizations (CSOs). The PATH country leads (also co-authors, named above) were the liaison with the appropriate authorities at community level.

When engaging with the local community, how did you ensure that the informed consent documents and other materials could be understood by local stakeholders?

Paragraph 3 of the Methods section provides details. Written documents and verbal explanations were provided to stakeholders in the appropriate local languages, and Paragraph 3 also documents how consent could be provided by illiterate caregivers.

Will the findings of the research be made available in an understandable format to stakeholders in the community where the study was conducted (e.g. via a presentation, summary report, copies of publications, etc.)? Please provide details of how this will be achieved.

The overall project findings are being made available in three journal articles (RCT, pre-post and cost studies) as well as more specialized publications on qualitative findings and specific health outcomes, currently in process. Outcomes were also presented as follows:  ***Improving Access to Tools that Detect Severe Illness*** meeting, which took place in-person in Dar es Salaam, Tanzania, **Tuesday – Thursday, June 11-13, 2024**. The event brought together TIMCI implementation teams from India, Kenya, Senegal and Tanzania, as well as the observer countries and national, regional, and global stakeholders working in child health. The participants reviewed key findings and learnings from the [TIMCI project](https://www.path.org/programs/market-dynamics/timci/), engaged in discussions on challenges and opportunities toward pulse oximetry and clinical decision support tools scale-up at the national level, and previewed key topics and trends related to pulse oximetry performance. A regular monthly newsletter was provided to members of the TIMCI Collaborator Group, as well as a primer on pulse oximetry <https://www.path.org/our-impact/resources/pulse-oximetry-primer/> and members of the TIMCI collaborator group were encouraged to distribute to relevant contacts including via social media. Individual country-level papers were provided to the country economists for Kenya, Senegal and Tanzania as requested, and country level economists were encouraged to use these materials for local stakeholders.
